# Hydrogen-Rich Saline Promotes Survival of Retinal Ganglion Cells in a Rat Model of Optic Nerve Crush

**DOI:** 10.1371/journal.pone.0099299

**Published:** 2014-06-10

**Authors:** Jing-chuan Sun, Tao Xu, Qiao Zuo, Ruo-bing Wang, Ai-qing Qi, Wen-luo Cao, Ai-jun Sun, Xue-jun Sun, Jiajun Xu

**Affiliations:** 1 Department of Anatomy, Second Military Medical University, Shanghai, PR China; 2 Graduates Management Unit, Second Military Medical University, Shanghai, PR China; 3 Department of Ophthalmology, Shanghai Jiaotong University Affiliated Shanghai First People's Hospital, Shanghai, PR China; 4 Department of Diving Medicine, Faculty of Naval Medicine, Second Military Medical University, Shanghai, PR China; Instituto Murciano de Investigación Biosanitaria-Virgen de la Arrixaca, Spain

## Abstract

**Objective:**

To investigate the effect of molecular hydrogen (H_2_) in a rat model subjected to optic nerve crush (ONC).

**Methods:**

We tested the hypothesis that after optic nerve crush (ONC), retinal ganglion cell (RGC) could be protected by H_2_. Rats in different groups received saline or hydrogen-rich saline every day for 14 days after ONC. Retinas from animals in each group underwent measurements of hematoxylin and eosin (H&E) staining, cholera toxin beta (CTB) tracing, gamma synuclein staining, and terminal deoxynucleotidyltransferase-mediated dUTP nick end labeling (TUNEL) staining 2 weeks post operation. Flash visual evoked potentials (FVEP) and pupillary light reflex (PLR) were then tested to evaluate the function of optic nerve. The malondialdehyde (MDA) level in retina was evaluated.

**Results:**

H&E, gamma synuclein staining and CTB tracing showed that the survival rate of RGCs in hydrogen saline-treated group was significantly higher than that in saline-treated group. Apoptosis of RGCs assessed by TUNEL staining were less observed in hydrogen saline-treated group. The MDA level in retina of H_2_ group was much lower than that in placebo group. Furthermore, animals treated with hydrogen saline showed better function of optic nerve in assessments of FVEP and PLR.

**Conclusion:**

These results demonstrated that H_2_ protects RGCs and helps preserve the visual function after ONC and had a neuroprotective effect in a rat model subjected to ONC.

## Introduction

Traumatic optic neuropathy (TON) is one of the most devastating causes of permanent visual loss and blindness [1]. Previous studies demonstrated that there are commonly two pathophysiology processes shared by retinal ganglion cells (RGCs) after optic nerve crush (ONC) [2], [3], which are necrosis and apoptosis. In 1999, Bien et al. found that after partial optic nerve injury, a fraction of RGCs underwent early necrosis, whereas others were subjected to delayed apoptotic death in the initial phase of RGC degeneration [2]. RGCs apoptosis was observed for up to 6 weeks, with most apoptotic cells in the first 2 weeks after ONC. Though research in this area is substantial, the specific mechanism underlying neural degeneration after ONC is not fully understood. Nevertheless, oxidative stress is now considered as one of the most important factors that mediate the process of apoptosis [4], [5].

Oxidative stress of cells is associated with the accumulation of reactive oxygen species (ROS), including superoxide anion (O_2_^−^), hydroxyl radical (•OH), hydrogen dioxide (H_2_O_2_), nitric oxide (NO), and peroxynitrite (ONOO^−^). Burst of ROS, especially •OH, breaks the balance between the oxidative and antioxidative system and irrevocably drives downstream signaling networks that lead to peroxidation of fatty acids or nucleic acids, elimination of protein cross-linking, and finally, apoptosis of cells [6]. Though H_2_ has long been regarded physiologically inactive within human body, Ohsawa et al. in 2007, reported that inhalation of hydrogen gas has an antioxidative effect to protect the brain against ischemia-reperfusion injury and stroke [7]. Their study showed that H_2_ is a special free radical scavenger, which selectively reduces •OH and NO•. In the following years, subsequent studies revealed that H_2_ is a potent antioxidant for many conditions implicated with oxidative stress, including ischemia-reperfusion injury [8]–[11], radiation induced injury [12], [13], metabolic disorder [14], [15], inflammation [16]–[18], and cancer [19]. H_2_ also shows neuroprotective effect due to its antioxidant property, such as spinal cord ischemia-reperfusion injury [20], neonatal hypoxia-ischemia[21], [22], neurodegenerative disorder [23]–[26], hearing loss [27], [28] and traumatic brain injury [29]. Furthermore, in optic neuropathy, Oharazawa et al. found H_2_-loaded eye drops reduced the retinal ischemia-reperfusion injury [30]. Wei et al. proved that H_2_-rich saline has neuroprotective effect against glutamate-induced excitotoxic injury of retinal neuron [31]. However, no studies have shown that H_2_ can protect RGCs in TON.

Considering the antioxidant property of H_2_ and the important role played by oxidative stress in TON, we launched this study to test the hypothesis that intraperitoneal administration of H_2_ saline could promote survival of RGCs and helps preserve the visual function after ONC.

## Methods

### Experimental groups

Fifty-four adult male Sprague-Dawley rats weighing 200–220 g were used in our experiments. Nine cages were used to raise the animals in a room with constant temperature and a 12-h light-dark cycle. The experiment was conducted according to the Guide for the Care and Use of Laboratory Animals recommended by the National Institutes of Health and approved by the Ethics Committee for Animal Experimentation of the Second Military Medical University. Animals were divided into three groups consisting of eighteen rats each: (1) H_2_ saline group: ONC in the left eye and hydrogen-rich saline (Department of Diving Medicine, Faculty of Naval Medicine, Second Military Medical University, Shanghai, China) administered intraperitoneally at 6:00 and 18:00 (5 ml/kg); (2) placebo group: ONC in the left eye and physiologic saline administered intraperitoneally at 6:00 and 18:00 (5 ml/kg); all the rats of H_2_ saline group and placebo group were administered intraperitoneal injection beginning at the time of ON crush and lasting for 2 weeks; (3) Sham group: sham surgery was performed in the left eye. The number of rats used in each experiment is summarized in Table 1.

**Table 1:** Summary of the rats used and the study design.

Method	Group	Number of rats
H&E, gamma synuclein, TUNEL	Sham	6
	Placebo	6
	H_2_	6
PLR, FVEP, CTB	Sham	6
	Placebo	6
	H_2_	6
MDA	Sham	6
	Placebo	6
	H_2_	6

H&E, hematoxylin and eosin staining; gamma synuclein, immunohistochemical staining of gamma synuclein; TUNEL, terminal-deoxy-transferase-mediated dUTP nick end labeling; PLR, pupillary light reflex; FVEP, flash visual evoked potential; CTB, FITC-conjugated cholera toxin B tracing; MDA, malondialdehyde assay.

### Optic nerve crush

Animals were anesthetized with ketamin (40 mg/kg body weight [BW]) and xylazine 4 mg/kg BW). With the aid of a binocular-operating microscope, a lateral canthotomy was performed in the superior orbital rim, and an incision of the conjunctiva lateral to the cornea was made in the left eye. The retractor bulbi muscle was then separated. Nerve crush at 2 mm posterior to the eyeball was performed with a Yasargil aneurysm clip for 20 seconds after the optic nerve was exposed [32]. The location of the optic nerve was about one-third away from the tip of the clip. Care was taken to avoid blood vessel damage around the optic nerve. The skin was then closed with sutures, and ampicillin was applied to the wound. In all cases we verified that the integrity of the retinal blood supply was not damaged using a direct ophthalmoscope. No operative complications such as corneal infection and cataract were observed.

### Hematoxylin and eosin (H&E) staining and observation 2 weeks post operation

Following sacrifice, six rats in each group were chosen randomly. The eyeballs were removed and processed for histopathology, including fixation in 4% paraformaldehyde, embedding in paraffin, and H&E staining using standard techniques. For H&E staining, slice thickness was set at 5 µm and 3 sections were selected from each eye in which optic nerve head area could be found when examined under a microscope. After rinsing with double-distilled water, they were dehydrated and mounted with mounting fluid. The retinas from each animal were imaged and Imaging-Pro-Plus software (Media Cybernetics Inc., Silver Spring, MD) was used to perform quantitative analysis of cell numbers. The cells in the RGC layer of each sample were counted in 10 high powered fields (HPF, 400×).

### Quantification of RGC survival after injury

RGC number was examined after 14 days since ONC. We used the anterograde tracer FITC-conjugated cholera toxin B (CTB-FITC; List Biological, Campbell, CA) to inject into the vitreous chamber of the eye two days before fixation and removal of the eyeball. The cornea and lens were removed after the eyes were enucleated, and then the remaining eye cups containing the retinas were soaked in 4% paraformaldehyde for 30 min and the eye cups were rinsed in PBS for 15 min soaked. The retinas were then extracted, flat-mounted, and cover slipped using 1∶1 glycerol/PBS [32]. We took the artificial method for counting, under a fluorescence microscope of 20× objective. We used the grid frame (0.5 mm×0.5 mm) to choose sample areas as the picture show. Then we counted the number of RCGs and calculated the density. RGC were sampled at the inner (1/6 retinal eccentricity), midperiphery (1/2 retinal eccentricity), and outer (5/6 retinal eccentricity) of each quadrant of the retinal flat mount, that is 12 photographs of each retina [33]. RGC densities were quantified by calculating the mean of the samples.

### Immunohistochemical staining of gamma synuclein of RGC survival after injury

Since a percentage of nuclei in RGC layer belong to displaced amacrine cells, we conducted gamma synuclein staining (RGCs specific immunohistochemistry staining) to assess the quantification of RGC survival. Immunohistochemical staining of gamma synuclein was done as described previously [34], Slice thickness was set at 5 µm and 3 sections were selected from each eye. Slides were rinsed and incubated with primary antibodies (1∶100) against gamma synuclein (Abcam, Cambridge, MA). And then a biotinylated rabbit secondary antibody (Vectastain Elite ABC kit; Vector Laboratories, Burlingame, CA) was placed on the sections and incubated. The RGCs were counted in 10 high powered fields (HPF, 400×).

### Terminal deoxynucleotidyltransferase-mediated dUTP nick end labeling (TUNEL) staining

TUNEL staining was performed on the remnant paraffin-embedded sections using the in-situ cell death detection kit (Roche Diagnostics Corp, Indianapolis, IN). According to standard protocols, the slides were incubated with 3% H_2_O_2_ for 5 min, rinsed, and then incubated in TdT buffer for 15 min at 22 Celsius degree. The TdT reaction mixture was added and incubated for 30 min at 37 Celsius degree After blocking with BSA and incubation with ABC, the TUNEL reaction was visualized by chromogenic staining with DAB (Sigma-Aldrich). To compare the TUNEL-positive cells in the three groups, we counted the TUNEL-positive cells in the RGC layer of each sample in 10 HPF (400×) 2 weeks after the ONC; every eye were averaged in three sections.

### Pupillary light reflex (PLR)

The method employed to record responses followed the previous study [35]. Rats were anesthetized initially with 3% halothane, 30% NO and 70% O_2_. A light anesthesia was maintained with 1% halothane, 30% NO and 70% O_2_ to avoid suppression of the PLR response detected with the use of higher dose of anesthetics [36]. A high-power light-emitting diode (LED) (MCWHL2/white, M625L2/red, M470L2/blue; Thorlabs, Inc., Newton, NJ) was set to provide illumination for a infrared-sensitive camera (PC164CEX-2; Supercircuits Inc., Austin, TX) that sent the image to computer. The responses elicited from the pupil could be observed directly on the screen of the computer. For stop-frame analysis, the computer recorded the data at a rate of 25 samples per second. A stimulus of 5 seconds duration followed by a recovery period of 60 seconds was used, which was sufficient for the returning of pupil to the baseline diameter. Prior to testing, animals were dark-adapted and anesthetized. The recorded responses were replayed on the video, frame by frame, and pupil images were analyzed using batch processing in Photoshop (CS6 extended; Adobe Systems, Inc.; San Jose, CA). This response was analyzed 6 times per animal. The relative amplitude was the difference between the diameter before light stimulation (baseline diameter) and the minimum diameter after stimulation, and expressed as a percentage of the baseline diameter.

### Flash visual evoked potentials (FVEP)

To evaluate the function of the optic nerve with evoked potential analyzing instrument (MK-NMD, Chongqing Ming Kang Sciences Company Limited, Chongqing, China), FVEP were measured 2 weeks after ONC. Following anesthesia, each animal was located in a stereotaxic frame. After cutting the skin and drilling a hole above visual cortex on the skull, an active electrode was placed on pachymeninx and a passive electrode was placed on the skin in the interorbital region. The settings were background illumination off, a single flash with rate of 2.0 Hz and 50 sweeps for a test. Only the latency and amplitude of the first negative N1 and positive P1 peaks of FVEP wave in the three groups were compared.

### Malondialdehyde (MDA) assay

The MDA amount in the obtained retinal homogenate was immediately evaluated using a colorimetric assay (MDA kit S0131, Beyotime Institute of Biotechnology, Shanghai, China.) as described previously [37].

### Statistical analysis

For all analysis, the analyzers were blinded to study group. All quantitative data were expressed as mean ± standard error of mean (SEM). We used the software of SPSS18.0 to determine statistical significance of difference between experimental and control samples. The results were analyzed by analysis of variance (ANOVA) and post hoc analysis with Tukey's post hoc comparisons. A probability level of P<0.05 was considered to be statistically significant.

## Results

### RGC density after optic nerve crush

**Figure 1:**
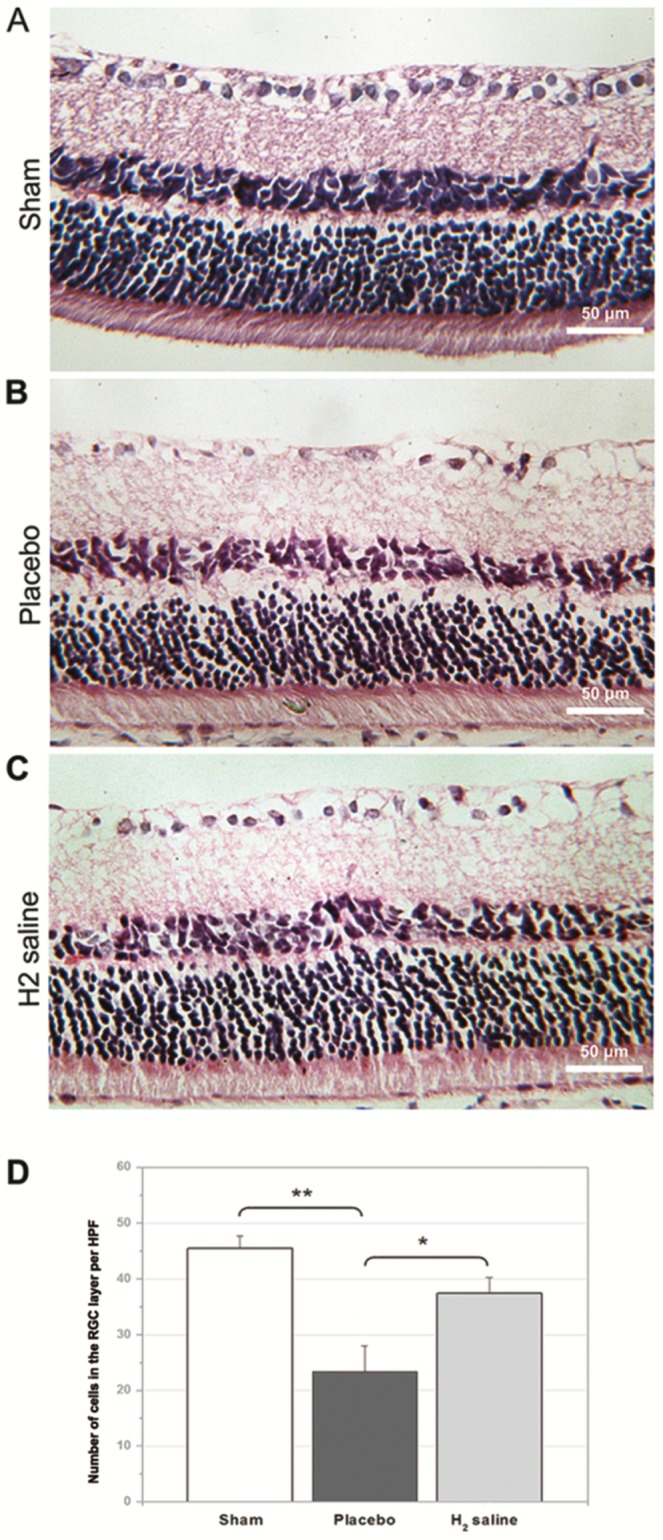
H&E staining of representative retinas. (A–C) H&E staining of retinal sections 2 weeks after ONC. (D) The number of cells in the ganglion cell layer of the H_2_-saline group was significantly higher than that of the placebo group (*p<0.05).

**Figure 2:**
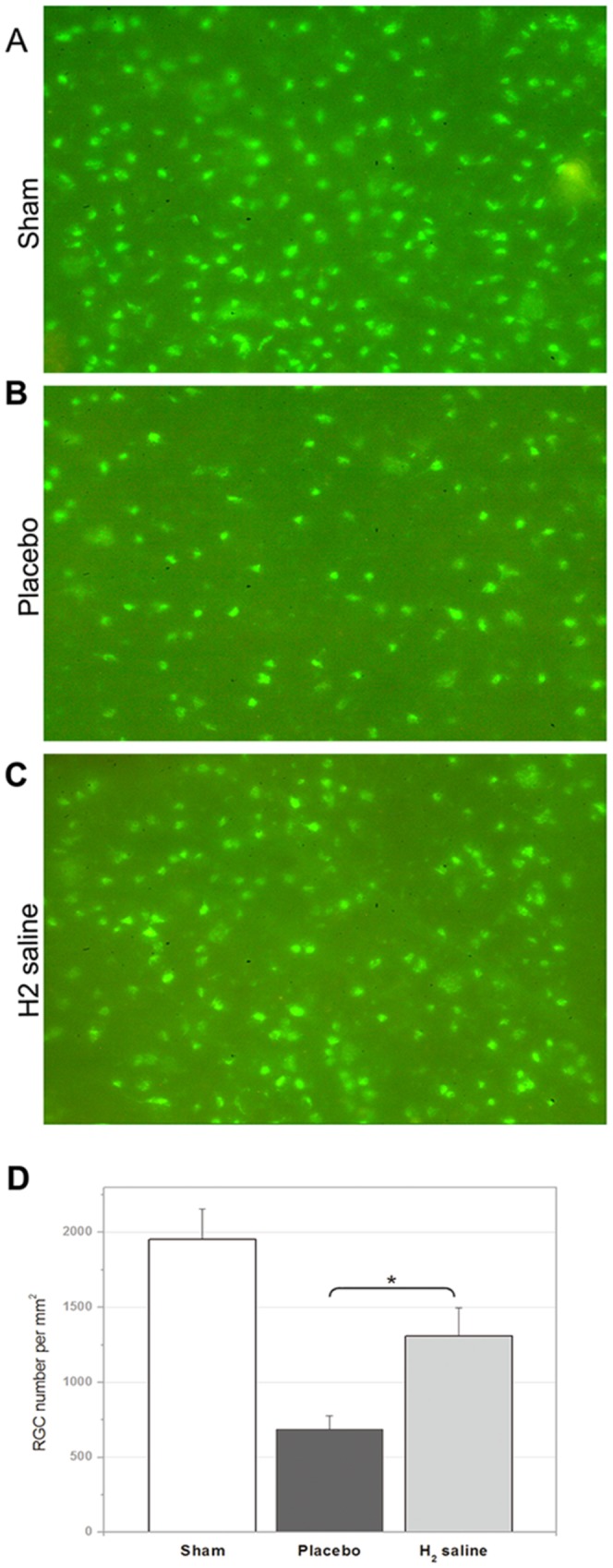
Morphometry of RGCs in representative flat-mounted retinas on the second day after the injection of the anterograde tracer CTB-FITC into the vitreous chamber of the eye. 2 weeks after ONC, RGC densities in the sham group (A), the Placebo group (B), and the H_2_ saline group (C). H_2_ saline treated showed a significant neuroprotective effect (the H_2_ saline group versus the placebo group, *p<0.05) (D).

**Figure 3:**
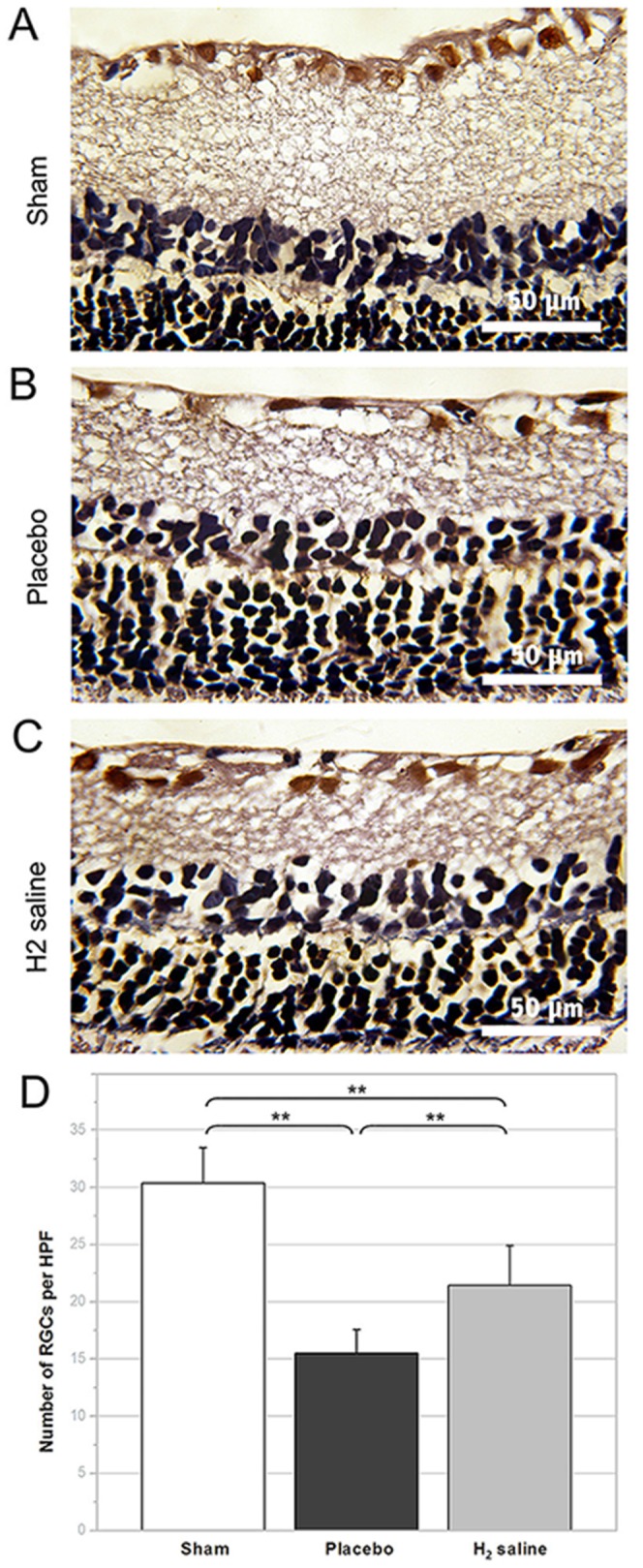
Gamma synuclein staining of representative retinas. (A–C) Gamma synuclein staining of retinal sections 2 weeks after ONC, gamma synuclein-positive RGCs are visualized in red brown colour. (D) The number of RGCs of the H_2_-saline group was significantly higher than that of the placebo group (**p<0.01).

Figure 1 shows representative samples of H&E staining from the H_2_ saline treated and untreated retinal explants. The number of H&E-stained cells in the ganglion cell layer of the sham group was 45.50±2.28 cells/HPF. There is no difference between sham group and H_2_ group (P = 0.246). More H&E-stained cells in the ganglion cell layer were observed in the H_2_ saline group than that in the placebo group demonstrating statistically significant difference between the survival rate of two groups (37.50±2.80 cells/HPF versus 23.40±4.57 cells/HPF; p<0.05; Fig. 1D). Moreover, the number of surviving RGCs was assessed by anterograde tracer FITC-conjugated cholera toxin B (CTB-FITC). The average RGC density in the retinas of the sham group was 1954.67±200.36 cells/mm^2^ VS H_2_ saline group 1308.68±190.54 cells/mm^2^ (P<0.05).The RGC densities in the H_2_ saline group and the placebo group decreased to 1308.68±190.54 cells/mm^2^, and 687.21±91.31 cells/mm^2^, respectively (p<0.05; Fig. 2D). Figure 3 shows representative samples of gamma synuclein staining from the H_2_ saline treated and untreated retinal explants. The number of gamma synuclein stained RGCs of the sham group was 30.39±3.11 cells/HPF. There is significant difference between sham group and H_2_ group (P<0.01). More gamma synuclein stained RGCs were observed in the H_2_ saline group than that in the placebo group demonstrating statistically significant difference between the survival rate of two groups (21.44±3.48 cells/HPF versus 15.50±2.09 cells/HPF; p<0.01; Fig. 3D).The results showed that the RGC survival rate was higher in the H_2_ saline group than that in the placebo group, which morphologically indicated that the H_2_ saline had a significant neuroprotective effect on rat's optic nerve after ONC.

### TUNEL staining

**Figure 4:**
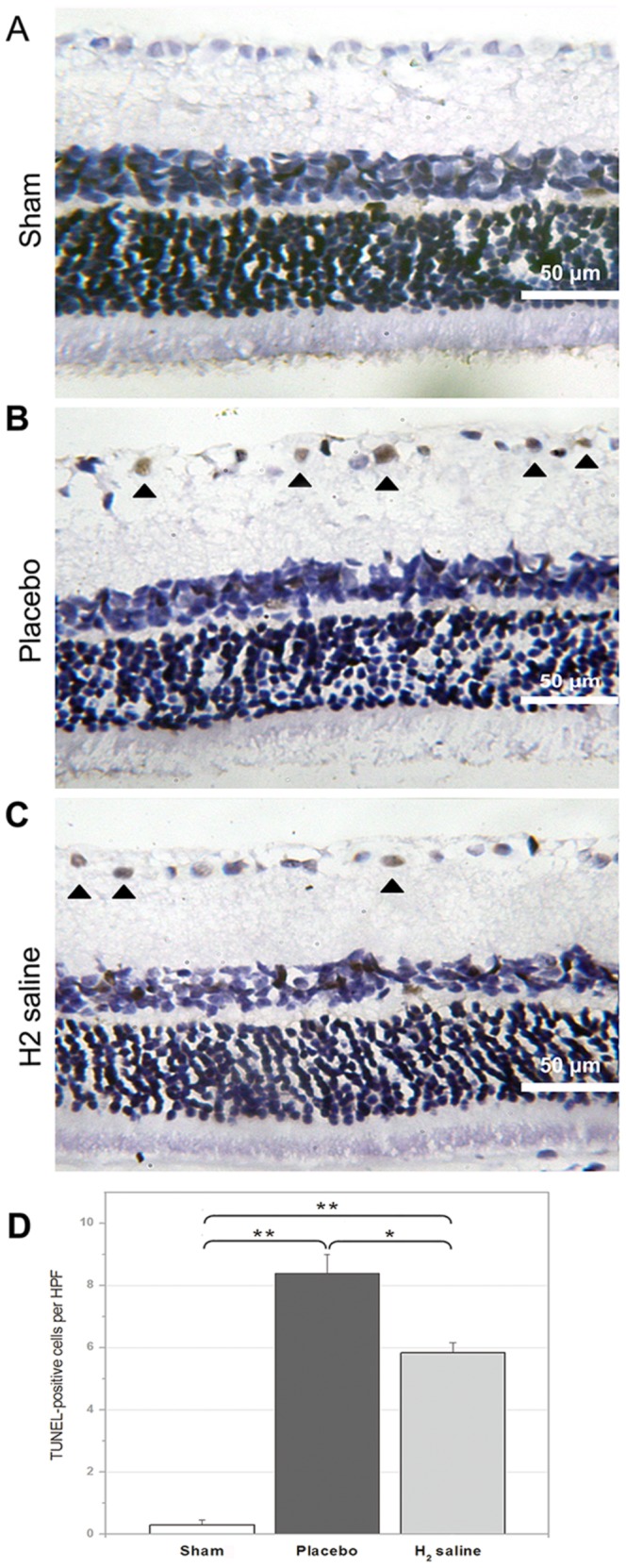
TUNEL staining of the representative retinas. TUNEL staining of retinal sections 2 weeks after ONC (A–D). Only the dark red-stained cells in the ganglion cell layer were counted as TUNEL-positive cells. There were few TUNEL-positive cells seen in the sham group (A). TUNEL-positive cells were observed in the placebo group (B) and the H_2_ saline group (C), and the chromatin was congregated on the cell border, with some cells forming apoptotic body morphology with a different shape. Note the significant increase (p<0.01) in the placebo group compared to the sham group, but the H_2_ saline group had fewer apoptotic cells than the placebo (*p<0.05) (D).

The TUNEL staining of retinal slice showed that the number of TUNEL-positive cells per HPF in the RGC layer was 0.29±0.16 cells in the sham group versus 5.83±0.33 cells in the H_2_ saline group (P<0.01), and 8.38±0.97 cells in the placebo group (p<0.05 versus the H_2_ saline group; Fig. 4). The results suggested that the neuroprotection effect of H_2_ in the rat model of ONC was conducted by reducing apoptosis of RGCs. The nuclei of RGCs were clearly stained in the placebo group, and few TUNEL-positive cells were seen in sham-operated rats (Fig. 4A). As shown in Figure 4, TUNEL-positive cell numbers were markedly increased in the RGC layer in the placebo group (Fig. 4C), and H_2_ saline reduced the number of TUNEL-positive cells (Fig. 4B, D), demonstrating that H_2_ saline had a significant antiapoptotic effect on RGCs after ONC.

### Pupillary light reflex

**Figure 5:**
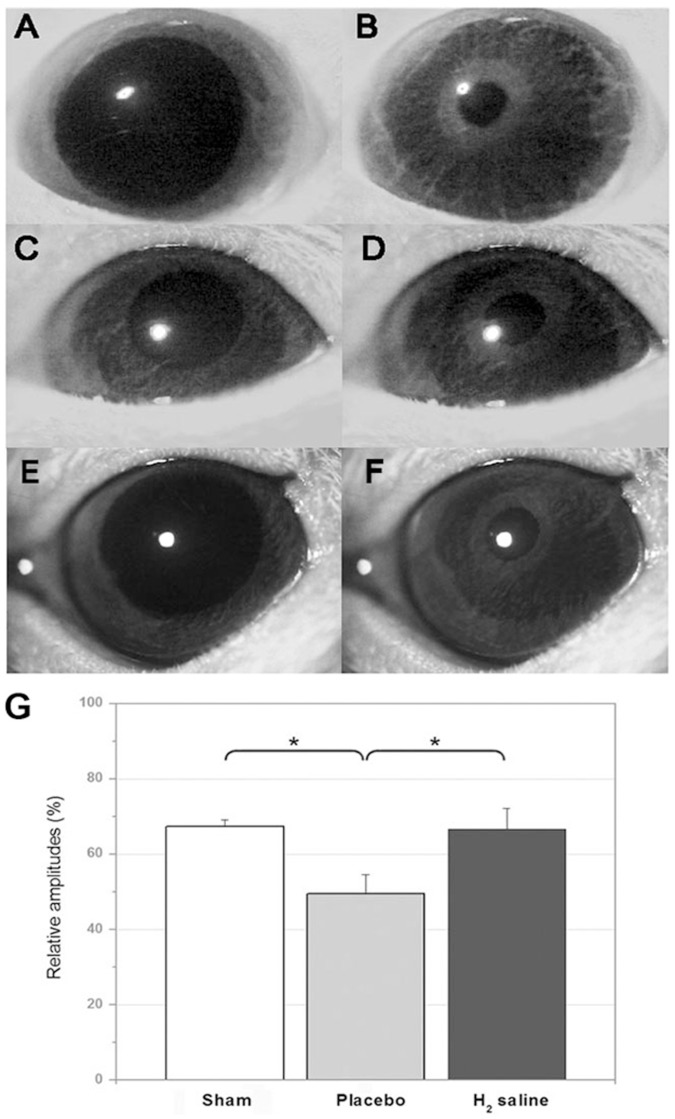
Video images of pupils recorded from rats. Pupils at the start (A, C and E) and end (B, D and F) of the stimulus. (A and B) Pupil recorded from a rat of the sham group, (C and D) from a rat of the placebo group, (E and F) from a rat of the H_2_ group. Pupil changes to the same light intensity. (G) The results of PLR assessment showed statistically higher relative amplitudes of rats in H_2_ groups than that of rats in placebo group(*p<0.05).

There are great individual differences in the initial size and diameter of the pupil in rats. However the contraction amplitude of the diameter after their pupillary light reflex is relatively stable [38]. Therefore, we use the amplitude as a parameter of PLR 2 weeks post operation. The mean relative amplitude of the H2 saline group was 67.29±1.79%, while the mean relative amplitude of the placebo group was 49.56±4.98% (Fig. 5, p<0.05 versus H_2_ saline group) and 68.44±7.89% in the sham group (p<0.05 versus placebo group). Six rats in each group were tested for pupillary response. As shown in Figure 5, the PLR measurements illustrate that the H_2_ saline group had significantly improved visual function compared to the placebo group.

### Flash visual evoked potentials

**Figure 6:**
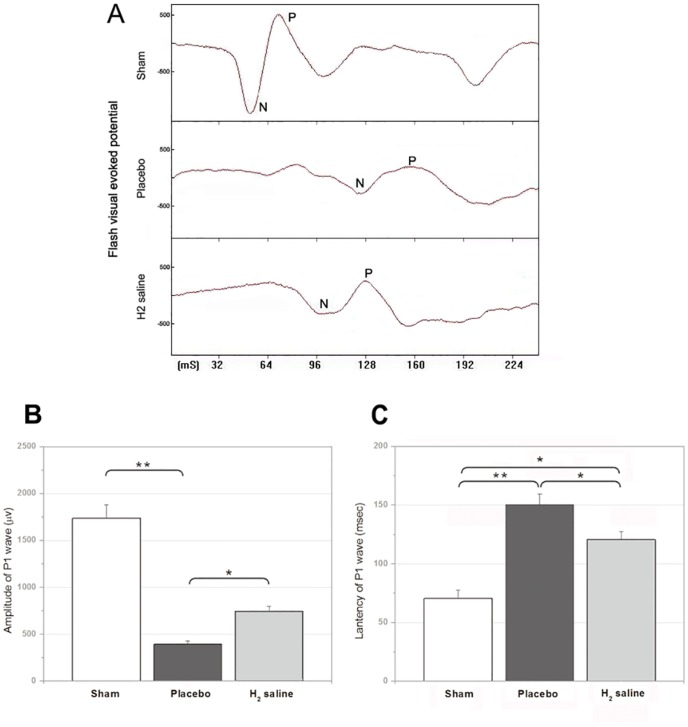
Flash visual evoked potential (FVEP) tracings. (A) Representative of FVEP tracings of each group. (B, C) The data of FVEP demonstrated a higher amplitude and a shorter latency in the H_2_ group than those in the placebo group and a prolonged latency and lower amplitude than those of the sham group (*p<0.05, **p<0.01, msec, milliseconds; ONC, optic nerve crush).

Flash visual evoked potentials were tested 2 weeks after surgery. The latency of the P1 wave was 70.56±7.18 msec (sham group), 120.74±6.79 msec (H_2_ saline group) and 150.34±9.33 msec (placebo group) (H_2_ saline group versus sham, p<0.05; H_2_ saline group versus placebo group, p<0.01; placebo group versus sham, p<0.01) (Fig. 6C). The amplitude of the P1 wave was 1737.44±142.24 µv (sham group), 741.23±56.34 µv (H_2_ saline group), and 394.34±36.56 µv (placebo group) (H_2_ saline group versus sham, p<0.05; placebo group versus sham, p<0.01) (Fig. 6B). The data of FVEP demonstrated a larger amplitude and a shorter latency in the H_2_ group than that in the placebo group.

### Malondialdehyde (MDA) assay

**Figure 7:**
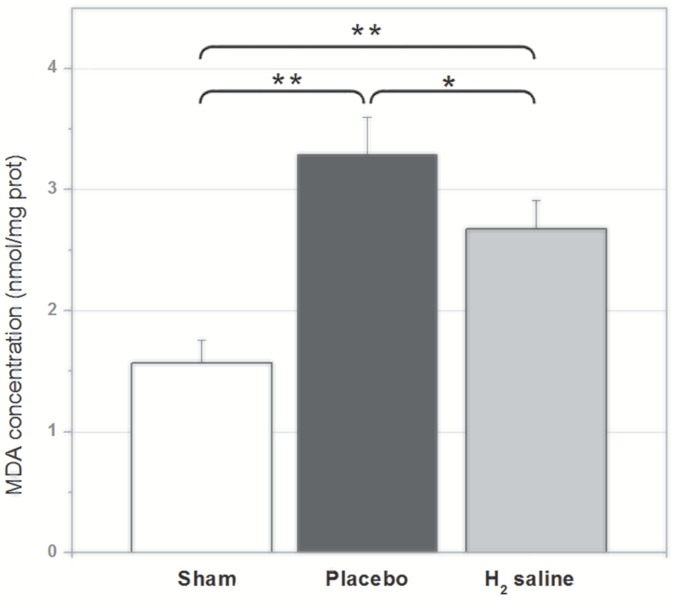
The MDA level in retina. The MDA level in retina of H_2_ group was significantly lower than that of placebo group but higher than that of the sham group (*p<0.05, **p<0.01).

The MDA level in retina of sham group was 1.57±0.36 nmol/mg prot (versus H_2_ group P<0.01). The MDA level in retina of H_2_ group and placebo group increased to 2.71±0.27 nmol/mg prot, 3.46±0.45 nmol/mg prot, respectively (P<0.05) as shown in Fig. 7. The results showed that the neuroprotective effect of H_2_ might be accomplished by suppressing oxidative neuronal stress.

## Discussion

This study demonstrated that H_2_ had a neuroprotective effect in a rat model subjected to ONC. The survival rate of RGCs was detected using H&E staining, gamma synuclein staining of retinal slice and CTB-FITC tracing of retina. The results of these assays provided morphologic evidence that H_2_ therapy promoted RGCs survival after ONC. TUNEL staining was used for labeling apoptotic cells, which showed less TUNEL-positive RGCs in H_2_ saline groups 2 weeks after ONC, suggesting that the neuroprotection effect of H_2_ in the rat model of ONC might be conducted by reducing apoptosis of RGCs. The level of MDA, the final product of lipid peroxidation, is an indicator of oxidative stress level. The results of MDA assay showed that the lipid peroxidation was decreased in H_2_ saline groups. To verify the visual function protection of H_2_, we assessed PLR, an involuntary reflex serving as an objective indicator of function. The results of PLR assessment showed statistically higher relative amplitudes of rats in H_2_ groups than that of rats in placebo group. The PLR is spatial summation over large areas of the retina whereas the FVEP is efficient at measuring the function of the central retina [39]. In this study, for FVEP assessment, shorter latency and higher amplitude of P1 wave in rats treated with H_2_ suggested better visual function preservation due to more axon survival of RGCs.

The mechanisms behind the protective effects of H_2_ remain unclear. One possible mechanism is via the prevention of apoptosis. Accumulation of ROS has been hypothesized to be one of the several mechanisms to cause the apoptotic process. Accumulation of ROS causes lipid peroxidation and release of arachidonic acids, which can cause damage to cells [40], [41]. In a study of Alzheimer's disease, hydrogen saline was demonstrated to decrease oxidative neuronal stress by reducing malondialdehyde (MDA, biomarker of lipid peroxidation) and 4-hydroxynonenal (HNE, biomarker of arachidonic acid peroxidation) [25]. Accumulation of ROS also causes glutamate-induced retinal excitotoxic injury [42]–[44]. Lihua Wei and his group demonstrated that the hydrogen-rich saline treatment may reduce the retinal neuron injury in guinea pig induced by glutamate [31]. The mechanism may involve promoting glutamate clearance, and further protecting the survival and function of glial cells.

Based on the ability of interrupting neuronal apoptosis, H_2_ therapy has been tested in many other studies. Ohsawa et al. in 2008 found that H_2_ can selectively reduce •OH and ONOO−, and protect the brain against ischemia–reperfusion injury [7]. In vivo study reported by Cai et al. demonstrated that H_2_ therapy offered neuroprotection by reducing HI-induced caspase-dependent apoptosis [21]. Lipid peroxidation level was decreased due to H_2_ administration, and neural apoptosis was thus reduced in the following study reported by Cai et al [22].

Instead of topical administration, the hydrogen-rich saline and physiologic saline were both administered intraperitoneally. Though contralateral eyes were used as control eyes in many studies [45], [46], we used the left eye of the rats in sham group as sham control and the left eye of the rats in placebo group as placebo control to avoid the potential influence to the contralateral eyes bought by general administration of hydrogen.

In this study, administration of hydrogen-rich saline was demonstrated promoting the survival of RGCs and preserving neurofunction of optic nerve against traumatic injury in ONC model. Though the mechanism underlying the neuroprotective effect of H_2_ is sophisticated and not yet fully understood, the result of TUNEL staining and MDA assay suggested that the neuroprotective effect of H_2_ showed in this study might be accomplished by decreasing oxidative neuronal stress and further reducing apoptosis of RGCs.

In conclusion, we demonstrated that hydrogen-rich saline provided neuroprotection to optic nerve, as indicated by assessment of RGC cell counts and functional assessment after ONC. The neuroprotective effects of H_2_ may involve antiapoptotic processes by decrease oxidative neuronal stress. Due to its efficacy, safety, and convenience, H_2_ saline may be a potential therapy for TON in the future.
